# Newborn Mice Vaccination with BCG.HIVA^222^ + MVA.HIVA Enhances HIV-1-Specific Immune Responses: Influence of Age and Immunization Routes

**DOI:** 10.1155/2011/516219

**Published:** 2011-04-12

**Authors:** Narcís Saubi, Eung-Jun Im, Raquel Fernández-Lloris, Olga Gil, Pere-Joan Cardona, Josep Maria Gatell, Tomáš Hanke, Joan Joseph

**Affiliations:** ^1^AIDS Research Unit, Hospital Clínic/IDIBAPS-HIVACAT, University of Barcelona, Calle Villarroel 170, 08036 Barcelona, Spain; ^2^MRC Human Immunology Unit, Weatherall Institute of Molecular Medicine, Oxford University and The John Radcliffe, Oxford OX3 9DS, UK; ^3^Unitat Tuberculosi Experimental, Institut “Germans Trias i Pujol”, Carretera del Canyet S/N, Badalona 08916, Barcelona, Spain

## Abstract

We have evaluated the influence of age and immunization routes for induction of HIV-1- and *M. tuberculosis*-specific immune responses after neonatal (7 days old) and adult (7 weeks old) BALB/c mice immunization with BCG.HIVA^222^ prime and MVA.HIVA boost. The specific HIV-1 cellular immune responses were analyzed in spleen cells. The body weight of the newborn mice was weekly recorded. The frequencies of HIV-specific CD8^+^ T cells producing IFN-*γ* were higher in adult mice vaccinated intradermally and lower in adult and newborn mice vaccinated subcutaneously. In all cases the IFN-*γ* production was significantly higher when mice were primed with BCG.HIVA^222^ compared with BCGwt. When the HIV-specific CTL activity was assessed, the frequencies of specific killing were higher in newborn mice than in adults. The prime-boost vaccination regimen which includes BCG.HIVA^222^ and MVA.HIVA was safe when inoculated to newborn mice. The administration of BCG.HIVA^222^ to newborn mice is safe and immunogenic and increased the HIV-specific responses induced by MVA.HIVA vaccine. It might be a good model for infant HIV and Tuberculosis bivalent vaccine.

## 1. Introduction

According to the last AIDS epidemic update released by UNAIDS on November 2010, an estimated 33.3 million people are currently living with HIV, and 2.6 million individuals became newly infected with the virus in 2009. Over the past year, the global AIDS epidemic killed 1.8 million people, and the number of children orphaned by AIDS was 16.6 million. It is estimated that 97% of these new infections occur in low- and middle-income countries, where ensuring universal access to antiretrovirals still represents an enormous challenge [1, 2]. In some sub-Saharan countries, the HIV prevalence among pregnant women can be over 30%. Approximately half of mother-to-child transmissions (MTCTs) are due to prolonged breastfeeding. Although around 35% of HIV-positive pregnant women are receiving antiretroviral therapy [1], reducing significantly mother-to-child transmission of HIV at delivery, the drugs have a high cost, have to be administrated after delivery, and maintained during the breastfeeding period, and the efficacy could be reduced due to emergence of resistant mutants. 

Neonatal immunity is immature compared to the adult immune system. CD8^+^ T-cell responses that may be critical to control intracellular pathogens including HIV and Mtb are inherently limited in human neonates. However, human and murine neonates generate functional Th1-type immune responses after infections with viruses or immunization with live-attenuated immunogens that deliver antigens into the cytoplasm of antigen presenting cells (APCs) [3]. Neonatal immunization could be the best approach to prevent infection or reduce the severity of HIV-related disease in these infants. Only two candidates vaccines designed to protect against breast milk HIV transmission have been studied in human infants. Therefore, there is an urgent need for a neonatal immunogen that generates HIV-specific immunity more rapidly. Recombinant BCG has been developed as a candidate neonatal vaccine vector against pertussis, measles, respiratory syncytial virus (RSV), and breast milk HIV transmission [3, 4].

BCG as a vaccine vector has a number of attractive features [5, 6]. BCG has a proven record of safety as a vaccine against tuberculosis from its use in over two billion individuals including neonates [7]. BCG infects and colonizes macrophages and dendritic cells, where it can survive and replicate for a long period of time. Through its persistence and potent adjuvantation by its cell wall components, it can induce long-lasting humoral and cellular immune responses. BCG can be given at or any time after birth and is not affected by maternal antibodies. Manufacturing of BCG-based vaccines is cheap and easy to purify. Finally, BCG is one of the most heat-stable vaccines in current use [8].

There is strong evidence supporting a role of cytotoxic T-lymphocytes (CTLs) in the containment of HIV replication, and several vaccine approaches are being pursued to elicit anti-HIV CTL responses [9–12]. One promising approach is that provided by *Mycobacterium bovis* Bacillus Calmette-Guérin (BCG) as a live recombinant bacterial vaccine vector. 

We have been developing a recombinant BCG-based HIV vaccine to induce protective cell-mediated responses. Our starting platform was based on a heterologous BCG prime and modified vaccinia virus Ankara (MVA) boost regimen delivering a common immunogen called HIVA, which is derived from consensus HIV-1 clade A Gag protein, that is, an immunogen derived from an HIV-1 strain prevalent in Central and Eastern Africa, and a string of CD8^+^ T-cell epitopes [13]. The recombinant *Mycobacterium bovis* Bacillus Calmette-Guérin (BCG) expressing HIVA immunogen has shown to be stable and to induce durable, high-quality HIV-1-specific CD4^+^ and CD8^+^ T-cell responses in BALB/c mice. Furthermore, when the recombinant BCG vaccine was used in a priming-boosting regimen with heterologous components, the HIV-1-specific responses provided protection against surrogate virus challenge, and the recombinant BCG vaccine alone protected against aerosol challenge with *M. tuberculosis* [14]. The BCG.HIVA^222^ vaccine candidate was vectored by a lysine auxotroph of BCG Pasteur strain that carried an *E. coli*-mycobacterial shuttle plasmid with a lysine A complementing gene and a weak promoter to regulate HIVA gene expression. This construction increases the plasmid stability *in vivo* and prevents heterologous gene expression disruption by genetic rearrangement [14, 15].

Infection with *Mycobacterium tuberculosis* kills about 2 million people each year [16]. *Mycobacterium bovis* Bacillus Calmette-Guérin (BCG) is the only licensed vaccine and protects significantly against childhood and miliary tuberculosis [17]. Globally, 80 percent of children are vaccinated with BCG, the majority of them at birth [18]. Thus, the development of a combined vaccine, which would protect neonates against tuberculosis and MTCT of HIV-1 through breastfeeding, is a logical effort in the fight against these two major global killers. Immunity against tuberculosis following neonatal BCG vaccination lasts 10 to 15 years and thus fails to protect adults from pulmonary disease [19, 20]. However, a 10-year immunity to HIV-1 would be an excellent start. 

We believe that the best hope to protect newborn children against MTCT of HIV-1 in developing countries is the development of a safe, effective, and affordable prophylactic vaccine, which would both reduce the adult burden of infection and protect neonates against vertical HIV-1 transmission. In the present study, we have evaluated the influence of age and immunizations routes for induction of HIV-1 and *M. tuberculosis*-specific immune responses after BALB/c mice immunization with BCG.HIVA^222^ prime and MVA.HIVA boost. The administration of BCG.HIVA^222^ to newborn mice is safe and immunogenic and increased the HIV-specific responses induced by MVA.HIVA vaccine. It might be a good model for infant HIV and Tuberculosis bivalent vaccine.

## 2. Materials and Methods

### 2.1. *E. coli*/mycobacterial Vector Expressing HIVA Antigen

The construction of *E. coli*/mycobacterial vector expressing HIVA antigen [13] was previously described [14]. Briefly, the coding sequence of the HIVA immunogen (consensus HIV-1 clade A Gag protein and a string of CD8^+^ T-cell epitopes) was fused to the *M. tuberculosis *nucleotides coding for the 19-kDa lipoprotein signal sequence in a PCR, and the chimeric gene was cloned into the pJH222 *E. coli*-mycobacterial shuttle plasmid (kindly provided by W. R. Jacobs Jr. and B. R. Bloom) as a HindIII-HindIII fragment under the control of the *M. tuberculosis α*-antigen promoter by using standard recombinant-DNA techniques.

### 2.2. Electroporation of Mycobacteria and Culture

A lysine auxotrophic strain of BCG was kindly provided by W. R. Jacobs Jr., B. R. Bloom, and T. Hsu. Mycobacterial cultures were grown in Middlebrook 7H9 broth medium or on Middlebrook agar 7H10 medium supplemented with Middlebrook ADC (albumin, dextrose, catalase) Enrichment (Difco) and containing 0.05% Tween 80 and 25 *μ*g of kanamycin/mL. The L-lysine monohydrochloride (Sigma) was dissolved in distilled water and used at a concentration of 40 *μ*g/mL. The plasmid pJH222HIVA was transformed into BCG lysine auxotrophic strains by electroporation. Cultures were grown to an OD of 0.9 (600 nm), pelleted at 3,000 rpm, washed twice by resuspension and centrifugation (3,000 rpm) in 10% glycerol at 4°C, and finally resuspended in 1/20th of the original culture volume of cold 10% glycerol. Then 100 *μ*L of the cold BCG suspension was mixed with plasmid DNA (50–500 ng) in a prechilled 0.2 cm electroporation cuvette and transformed using the BioRad Gene Pulser electroporator at 2.5 kV, 25 mF, and 1,000 *Ω*. After electroporation 1 mL of 7H9 medium (Difco), supplemented with albumin-dextrose-catalase (ADC, Difco) and containing 0.05% Tween 80 (SIGMA), was added and incubated at 37°C for 12 hours before plating onto Middlebrook agar 7H10 medium (Difco) supplemented with ADC (Difco) and containing 0.05% Tween 80 (SIGMA) and 25 *μ*g/mL of kanamycin.

### 2.3. Western Blot Analysis

BCG transformants were grown to mid-logarithmic phase in liquid 7H9 (Difco) medium supplemented with albumin-dextrose-catalase (ADC, Difco) and containing 0.05% Tween 80 and kanamycin (25 *μ*g/mL). rBCG cultures were centrifuged at 3,000 rpm for 10 minutes at 4°C. Pellets were washed twice in PBS plus 0.02% Tween 80 and resuspended in 1 mL of extraction buffer (50 mM Tris-HCl pH 7.5, 5 mM EDTA, 0.6% sodium dodecyl sulfate), and 5 *μ*L of 100x protease inhibitor cocktail (1 mg/mL aprotinin, 1 mg/mL E-64, 1 mg/mL leupeptin, 1 mg/mL pepstatin A, 50 mg/mL pefabloc SC, and 10 mL DMSO) was added. Cells were sonicated for 4 minutes on ice with a Branson Sonifier at output control seven, duty cycle 50%. Extracts were centrifuged at 13,000 rpm for 10 minutes at 4°C, and supernatants were collected. Proteins were separated on 15% SDS-polyacrylamide gel. HIVA protein was detected using anti-Pk antibodies with an ECL kit (Amersham International).

### 2.4. Mice

Six BALB/c female mice per group, 7 weeks old (adult) and 7 days old (newborn), were used. The animal experiment was approved by the local ethical committee for animal experiments from the University of Barcelona and strictly conformed to Catalan Animal Welfare legislation.

### 2.5. Immunization and Isolation of Splenocytes

Adult mice were immunized intradermally with 2 × 10^6^ CFU of BCG.HIVA^222^ or subcutaneously with 10^6^ CFU of BCG.HIVA^222^. Newborn mice were immunized subcutaneously with 2 × 10^6^ CFU of BCG.HIVA^222^. Adult and newborn mice were boosted 14 weeks after BCG inoculation with 10^6^ PFU of MVA.HIVA intramuscularly, and 3 weeks later the animals were sacrificed. The animals were sacrificed by cervical dislocation. The newborn mice were weighted weekly for the safety testing of the vaccine. On the day of sacrifice, spleens were removed and pressed individually through a cell strainer (Falcon) with a 5 mL syringe rubber plunger. Following the removal of red blood cells with red blood cell lysing buffer (Sigma), splenocytes were washed and resuspended in lymphocyte medium (RPMI 1640 supplemented with 10% fetal calf serum (FCS) and penicillin-streptomycin, 20 mM HEPES, and 15 mM *β*-mercaptoethanol) at a concentration of 2 × 10^7^ cells/mL.

### 2.6. Peptides

For assessing the immunogenicity of HIVA in the BALB/c mice, the following peptides were used: H-2D^d^-restricted epitope P18-I10 (RGPGRAFVTI), herein designated epitope H. The PPD (Purified Protein Derivative, Statens Serum Institute, Copenhagen) was used to assess the immunogenicity induced by *Mycobacterium bovis* BCG.

### 2.7. In Vitro Killing Assay

P815 cells (mouse lymphoblast-like mastocytoma cell line) were used as target cells. They were incubated (pulsed) without or with 2 *μ*g of H peptide/mL in R10 at 37°C in 5% CO_2_ for 90 min and washed three times. Target cells not pulsed with peptide were labeled with 5-(and-6)-(((4-chloromethyl)benzoyl)amino)tetramethyl rhodamine (CMTMR; Molecular Probes) only, while peptide-pulsed target cells were labeled with carboxyfluorescein diacetate succinimidyl ester (CFSE, Molecular probes). Briefly, H peptide-pulsed P815 cells resuspended in phosphate-buffered saline (PBS) at 2 × 10^7^ cells/mL were incubated with 80 nM CFSE in the dark at room temperature for 10 min, followed by the quenching of the reaction with an equal volume of FCS and three washing steps with R10. H peptide-pulsed cells (and similarly control nonpulsed cells) were then resuspended in R10 at 2 × 10^7^ cells/mL and incubated with 10 *μ*M CMTMR at 37°C for 15 min and in fresh R10 only for a further 15 min. Cells were washed 3 times as previously described. Finally, pulsed and non pulsed P815 cells were mixed at 1 : 1 ratio. Splenocytes obtained from vaccinated mice and expanded for 5 days in lymphocyte medium containing 2 *μ*g/mL of H peptide were harvested and counted. In a 96-well round bottom plate, 200 *μ*L of a suspension containing 10^6^ expanded splenocytes were placed in duplicate wells, and two 2-fold dilutions in R10 were performed. On top, 100 *μ*L of a suspension contained the mixture of pulsed and unpulsed P815 cells, 10^4^ of each (at 100 : 1, 50 : 1, and 25 : 1 effector : target cells ratio). After a minimum of 5 h of reaction, the cells were fixed and analyzed by flow cytometry. Cytotoxicity was calculated using the following formula: adjusted percentage of surviving cells = 100 × (percentage of surviving peptide-pulsed cells/mean percentage of surviving unpulsed cells). Next, the percentage of specific lysis was calculated as follows: percentage of specific lysis = 100 − adjusted percentage of surviving cells [21]. The specific CTL activity was assessed between the BCGwt or BCG.HIVA^222^ primed mice.

### 2.8. Intracellular Cytokine Staining

Two million splenocytes were added to each well of a 96-well round-bottomed plate (Falcon) and pulsed with 2 (CD8 epitopes) to 5 *μ*g/mL (CD4 epitopes) peptides or 5 *μ*g/mL PPD tuberculin (Statens Serum Institut, Copenhagen, Denmark) together with antibodies against lysosomal-associated membrane proteins anti-CD107a-FITC/anti-CD107b-FITC (BD Biosciences) [22] and kept at 37°C, 5% CO_2_ for 90 minutes, followed by the addition of GolgiStop (BD Biosciences) containing monensin. After a further 5-hour incubation, reaction was terminated, and the cells were washed with FACS wash buffer (PBS, 2% FCS, 0.01% Azide) and blocked with anti-CD16/32 (BD Biosciences) at 4°C for 30 minutes. All subsequent antibody stains were performed using the same conditions. Cells were then washed and stained with anti-CD8-PerCP or anti-CD4-PerCP (BD Biosciences), washed again, and permeabilized using the Cytofix/Cytoperm kit (BD Biosciences). Perm/Wash buffer (BD Biosciences) was used to wash cells before staining with anti-IL-2-FITC, anti-TNF-*α*-PE, and anti-IFN-*γ*-APC (BD Biosciences). Cells were fixed with CellFIX (BD) and stored at 4°C until analysis.

### 2.9. Ex Vivo IFN-*γ* ELISPOT Assay

The ELISPOT assay was performed using the Mabtech IFN-*γ* ELISPOT kit according to the manufacturer's instructions. The ELISPOT plates (Millipore ELISPOT plates) were coated with purified anti-mouse IFN-*γ* capture monoclonal antibody diluted in PBS to a final concentration of 5 ug/mL at 4°C overnight. The plates were washed once in R10 and blocked for 2 h with R10. A total of 5 × 10^5^ fresh splenocytes were added to each well, stimulated with 2 *μ*g/mL of the H peptide or 5 *μ*g/mL of PPD for 16 h at 37°C, 5% CO_2_, and lysed by incubating twice with deionized water for 5 minutes. Wells were then washed 3x with PBS 0.05% Tween 20, incubated for 2 h with a biotinylated anti-IFN-*γ* mAb diluted in PBS 2% FCS to a final concentration of 2 ug/mL, washed 3x in PBS 0.005 Tween 20, and incubated with the Streptavidin-Alkaline Phosphatase-conjugate in PBS 2% FCS. Wells were washed 4x with PBS 0.005 Tween 20 and 2x with PBS before incubating with substrate solution (Alkaline Phosphatase Substrate, BioRad). After 5–10 minutes, the plates were washed with tap water, dried, and the resulting spots counted using an ELISPOT reader (AIC).

### 2.10. Fluorescence-Activated Cell Sorter Analysis

All chromogen-labeled cells were analyzed in a Becton Dickinson FACScalibur, using the CellQuest software for acquisition (BD Biosciences) and the Flow-Jo software (Tri-Star) for analysis.

### 2.11. Statistical Analysis

Data are the means ± SEM (standard error or the mean) for six mice per group. Statistical significance was determined by ANOVA (* = *P* < .05; ** = *P* < .01; *** = *P* < .001).

## 3. Results

### 3.1. Recombinant *Mycobacterium bovis* BCG Expressing HIV-1 Clade A Immunogen

**Figure 1:**
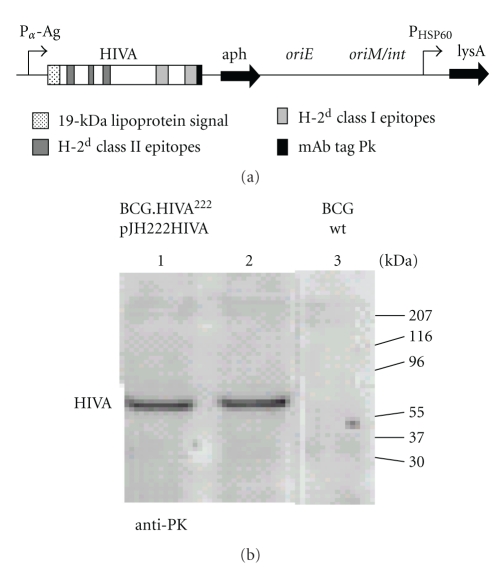
Expression of the HIVA immunogen from BCG.HIVA^222^. (a) A synthetic GC-rich *HIVA *gene consisting of consensus was fused to the 19-kDA lipoprotein signal sequence and inserted into episomal pJH222 *E. coli*-mycobacterium shuttle plasmid. This contains a kanamycin resistance gene (*aph*) and complementing *lysA *genes and *E. coli *origin of replication (*oriE*). In addition, pJH222 contains mycobacterial origin of replication (oriM). BALB/c mice T-cell and mAb Pk epitopes used in this paper are depicted. P*α*-Ag, *M. tuberculosisα*-antigen promoter; PHSP60, heat shock protein 60 gene promoter. (b) Western blotting of lysates of BCG.HIVA^222^ containing the pJH222HIVA (lanes 1 and 2) and BCG wild type (lane 3; negative control) is shown. HIVA was detected using the anti-Pk mAb followed by HRP-protein A and ECL.

HIVA immunogen consists of consensus HIV-1 clade A gag p24/p17 domains coupled with a string of CD8^+^ T-cell epitopes and monoclonal antibody (mAb) tag Pk [13]. The HIVA gene was synthesized utilizing humanized GC-rich codons, which are similar to those used by mycobacteria [23–25]. The HIVA open-reading frame was fused at its 5′ end to nucleotides coding for the 19-kDa lipoprotein signal sequence, which facilitates expression of foreign proteins in the mycobacterial membrane and was shown to increase the foreign protein immunogenicity [26]. To facilitate the preclinical development of candidate vaccines, the HIVA immunogen contains an immunodominant H-2D^d^-restricted epitope P18-I10 [27], here designated also as H epitope. In addition, it also contains at least three other subdominant H-2D^d^ epitopes recognized by CD8^+^ T cells including epitope P and three CD4^+^ T-helper epitopes (unpublished). The chimeric 19-kDa signal sequence-HIVA gene was expressed from *Escherichia coli*/mycobacterium shuttle plasmid pJH222 under the control of the *M. tuberculosisα*-antigen promoter (Figure 1(a)). pJH222 is a low-copy replicative episomal vector and contains mycobacterial origin of replication (*oriM*). It contains also an expression cassette encoding kanamycin resistance (*aph*), *E. coli* origin of replication (*oriE*), and a wild-type lysine A-complementing gene for the vector maintenance (*lysA5*) in the BCG auxotroph. Recombinant pJH222.HIVA was transformed into lysine auxotroph of *M. bovis* BCG host strain Pasteur ∆lysA5::res [28]. Expression of the full-size chimeric 19-kDa signal sequence-HIVA protein of *M_r_* 65 kDa was confirmed on a Western blot of whole transformed mycobacterial cell lysates using anti-Pk mAb (Figure 1(b)).

### 3.2. BCG.HIVA^222^ Prime and MVA.HIVA Boost Elicited Functional HIV-1-Specific CD8^+^ T-Cell Responses

**Figure 2:**
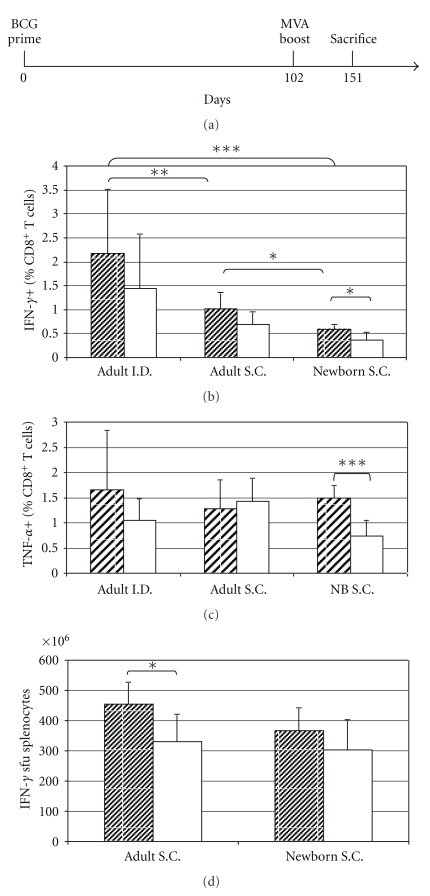
Effect of BCG.HIVA^222^ priming on the induction of HIV-1-specific CD8^+^ T cells. (a) Mice were immunized with 2 × 10^6^ cfu (adult mice I.D. or neonates S.C.) or 10^6^ cfu (adult mice S.C.) of BCG.HIVA^222^ and subsequently boosted 14 weeks later with 10^6^ pfu of MVA.HIVA by i.m. route. (b and c) Analysis of IFN-*γ* and TNF-*α* vaccine-elicited CD8^+^ T cells as generated for each vaccination group by using the P18I10 epitope. The frequencies of CD8^+^ T cells producing IFN-*γ* or TNF-*α* are shown. Data are presented as means ± standard deviation (SD; *n* = 6). (d) Elicitation of specific T-cell responses was assessed in an IFN-*γ* ELISPOT assay using the immunodominant P18I10 CD8^+^ T-cell epitope peptide. The mean (± SEM) sfu per 10^6^ splenocytes for each group of mice (*n* = 6 per group) is shown. BCG.HIVA^222^ primed mice: striped bars. BCGwt primed mice: white solid bars. * = *P* < .05; ** = *P* < .01, *** = *P* < .001.

We have demonstrated in previous studies with BALB/c mice that BCG.HIVA^222^ can both prime novel and boost preexisting MVA.HIVA-elicited HIV-1-specific CD4^+^ and CD8^+^ cellular immune responses of high quality upon antigenic reexposure [14]. In this study we have evaluated the effect of BCG.HIVA^222^ priming using different routes and mice age (adult and newborn) on the induction of HIV-1-specific T-cell responses after BALB/c mice immunization with BCG.HIVA^222^ prime and MVA.HIVA boost. The immunogenicity readout was focused on the P18I10 epitope, an immunodominant CTL epitope derived from HIV-1 Env and H-2D^d^ restricted [27], here mentioned as H, which was fused to HIVA immunogen to evaluate the immunogenicity in mice (Figure 1(a)). On day 0, mice were immunized with rBCG with the episomal plasmid or BCG wild type, and on day 102 the animals received a booster dose with MVA.HIVA. On day 151, the mice were sacrificed, and the functional specific T cells in response of peptide stimulation were measured by intracellular cytokine staining (ICS) and ELISPOT assays (Figure 2(a)). We have observed in adult and newborn mice that BCG.HIVA^222^ prime and MVA.HIVA boost induced higher frequencies of H-specific CD8^+^ splenocytes producing IFN-*γ* and TNF-*α* compared with the BCG wild-type priming and MVA.HIVA boost in two analyses performed (Figures 2(b), 2(c), and 2(d)). Overall, the proportions of HIV-1-specific T cells producing IFN-*γ* and TNF-*α* were higher in adult mice compared with newborn mice. When adult mice were vaccinated intradermally, the BCGwt priming elicited 1.45% of CD8^+^ T cells producing IFN-*γ*, in comparison with 2.18% when the priming was performed with BCG.HIVA^222^. We have detected the same pattern but lower magnitude when adult mice were immunized subcutaneously, 0,69% and 1,02%, respectively. When the newborn mice were primed with BCGwt or BCG.HIVA^222^ subcutaneously, the results were 0.37% and 0.59%, respectively (*P* < .05). The frequency of specific CD8^+^ splenocytes producing IF-*γ* was twofold higher (*P* < .01) when adult mice were primed with BCG.HIVA^222^ intradermally compared with subcutaneously and nearly 4-fold higher when compared with newborn mice (*P* < .001) (Figure 2(b)). The proportion of specific CD8^+^ splenocytes producing TNF-*α* was higher when mice were inoculated with BCG.HIVA^222^ (1.66% for adult and I.D. route and 1.50% for Newborn and S.C. route) in comparison with those inoculated with BCGwt (1.05% and 0.75%, resp.). In newborn mice, this difference was significant (*P* < .001). In contrast, the proportion was slightly higher in adult mice using BCGwt priming subcutaneously (1.42%) compared with BCG.HIVA^222^ priming (1.29%) (Figure 2(c)). 

The capacity of splenocytes from vaccinated mice to secrete IFN-*γ* was tested also by ELISPOT assays. The splenocytes secreted IFN-*γ* after overnight stimulation with the dominant CD8^+^ T-cell P18I10 epitope peptide. Representative results obtained with splenocytes from mice primed with BCG.HIVA^222^ or BCG wild type and boost with MVA.HIVA are shown in Figure 2(d). In adult and newborn mice the frequency of specific cells secreting IFN-*γ* was higher in mice primed with BCG.HIVA^222^ (455 and 367 spot forming units, (sfu)/10^6^ splenocytes for adult and newborn mice, resp.) compared with BCG wild-type (329 and 303 sfu/10^6^ splenocytes for adult and newborn mice, resp.). Among adult mice, the difference was significant (*P* < .05).

### 3.3. Priming BCG.HIVA^222^ Route and Age Affects the Level and Quality of CD8^+^ T-Cell Responses to an Immunodominant Epitope

**Figure 3:**
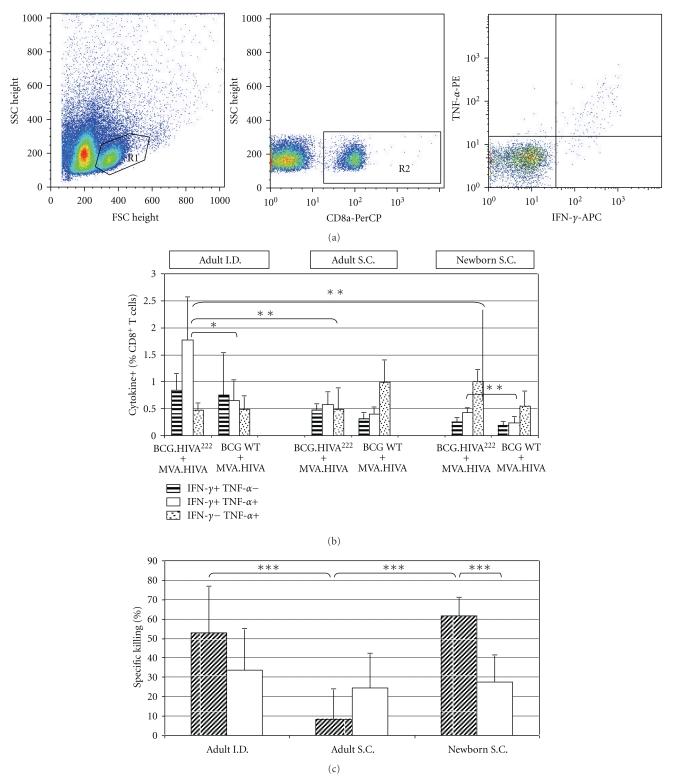
Induction of multifunctional HIV-1-specific CD8^+^ T cells by the BCG.HIVA^222^ prime and MVA.HIVA boost regimen. Analysis of bifunctional vaccine-elicited CD8^+^ T cells using the P18I10 peptide stimulation. (a) Example of dot blots and region selection for analysis. Region selected to analyze the splenocytes in the SSC-FSC dot-blot presentation (left), the CD8+ splenocytes selection (middle) and the IFN*γ* and TNF-*α* staining of the CD8^+^ splenocytes (right). (b) This panel shows the frequencies of CD8^+^ T cells producing IFN-*γ* and/or TNF-*α*. Data are presented as means ± SEM. (c) *In vitro* analysis of the CTL activity using peptide-pulsed target cells. BCG.HIVA^222^ primed mice: striped bars. BCGwt primed mice: white solid bars. * = *P* < .05; ** = *P* < .01, *** = *P* < .001.

We have evaluated the influence of route inoculation and mice age on the level and quality of CD8^+^ T-cell responses induced after mice immunization with BCG.HIVA^222^ prime and MVA.HIVA boost. The immunization schedule is described in Figure 2(a). The quality of vaccine-elicited CD8^+^ T cells was monitored by bifunctional analysis and *in vitro* killing assay for CTL activity. First, the frequencies of bifunctional IFN-*γ* and TNF-*α* T cells specific for the H epitope were assessed. We found that the magnitude of the bifunctional response was higher when the I.D. route was used for BCG.HIVA^222^ priming in adult mice compared with S.C. route in adult mice (*P* < .01), and S.C. route in newborn mice (*P* < .01). In addition, when the intradermal inoculation was performed, the proportion of CD8^+^ splenocytes producing IFN-*γ* and TNF-*α* was more than 2,5-fold higher in BCG.HIVA^222^ primed mice (1.77%) compared with BCG wild-type primed mice (0.65%) (*P* < .05). On the other hand, we observed the same trend but in lower magnitude when adult mice were inoculated subcutaneously with BCG.HIVA^222^ (0.58%), compared with BCG wild type (0.40%). The magnitude of the bifunctional response was lower in newborn mice than in adult mice. Besides that, the frequency of specific CD8^+^ splenocytes producing IFN-*γ* and TNF-*α* was higher in BCG.HIVA^222^ primed mice than in BCG wild-type primed mice (0.43 and 0.24%, resp., (*P* < .05) (Figure 3(b)). Second, the cytotoxic activity of the BCG.HIVA^222^-MVA.HIVA-elicited CD8^+^ T cells was also assessed by in vitro killing assay. Splenocytes were cultured and stimulated with the P18I10 peptide for five days and evaluated as effector cells. These effector cells were able to kill efficiently P815 target cells pulsed with the P18I10 peptide. The frequency of specific killing was higher in newborn mice than in adult mice. In newborn mice the proportion of specific CTL activity was higher after BCG.HIVA^222^ priming (61%) compared with BCG wild-type priming (27%), subcutaneously, *P* < .001. In adult mice the proportion of specific CTL activity was also higher after BCG.HIVA^222^ priming (53%) compared with BCG wild-type priming (34%) intradermally. In contrast, when adult mice were vaccinated by S.C. route, the highest frequency of specific killing was obtained when BCGwt priming was used (24.4%), compared with BCG.HIVA^222^ priming (8.2%) (Figure 3(c)). The frequency of specific killing was clearly higher in adult mice primed with BCG.HIVA^222^ I.D. (*P* < .001) and Newborn mice primed with BCG.HIVA^222^ S.C. (*P* < .001) in comparison with adult mice inoculated S.C.

### 3.4. BCG.HIVA^222^ Elicited PPD-Specific Responses in Mice

**Figure 4:**
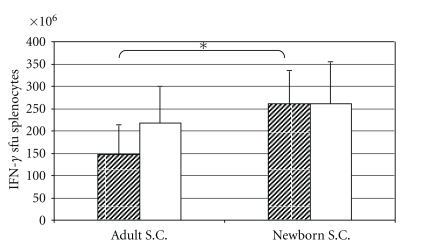
PPD-specific T-cell responses elicited by BCG.HIVA^222^. Immune responses to BCG were assessed in an *ex vivo* IFN-*γ* ELISPOT assay using PPD as the antigen. The mean (± SEM) sfu per 10^6^ splenocytes for each group of mice (*n* = 6 per group) is shown. BCG.HIVA^222^ primed mice: striped bars. BCGwt primed mice: white solid bars. * = *P* < .05.

The BCG-specific immune responses were assessed following the vaccine regimen consisting of BCG.HIVA^222^ prime and MVA.HIVA boost as described in Figure 2(a). The capacity of splenocytes from vaccinated mice to secrete IFN-*γ* was tested by ELISPOT assays. The splenocytes secreted IFN-*γ* after overnight stimulation with the PPD antigen. The frequencies of specific cells secreting IFN-*γ* was higher in newborn mice than in adult mice (261 sfu and 147 sfu/10^6^ splenocytes, resp., *P* < .05). On the other hand, in newborn mice, the proportion of specific cells secreting IFN-*γ* was identical after BCG.HIVA^222^ priming compared with BCG wild-type priming. (261 and 261 sfu/million splenocytes). However, in adult mice, the proportion of specific cells secreting IFN-*γ* was lower after BCG.HIVA^222^ priming compared with BCG wild-type priming (147 and 218 sfu/million splenocytes, resp.) (Figure 4).

### 3.5. BCG.HIVA^222^ Prime and MVA.HIVA Boost Regimen Was Safe in Newborn Mice

**Figure 5:**
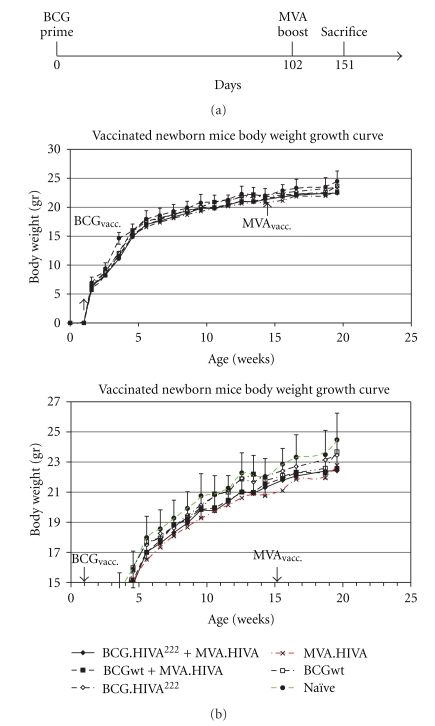
Body weight curve after newborn mice vaccination with BCG.HIVA^222^ (SC) and MVA.HIVA (IM). (a) Newborn mice were either left immunized or immunized with 2 × 10^6^ cfu of BCG wild type or BCG.HIVA^222^ by subcutaneous route and subsequently given a booster dose of 10^6^ pfu of MVA.HIVA as indicated in panel A. (b and c) The body weight was weekly recorded, and the body weight mean (± SEM) for each group of mice (*n* = 6 per group) is shown. Two different scales are shown so that the full evolution of the body weight is seen (b), and the *y*-axes zoomed to the 15–25 g interval, so that the group differences are better seen (c).

Six newborn mice (7 days old) per group were either immunized or left unimmunized with 2 × 10^6^ cfu of BCG wild type or BCG.HIVA^222^ subcutaneously route and subsequently given a booster dose of 10^6^ pfu of MVA.HIVA via intramuscular as described in Figure 2(a). As shown in Figure 5, the body weight was weekly monitored and recorded. All vaccine combinations were analyzed, to depict any possible adverse event due to vaccination and monitored by body weight lost. For rigorous safety assessment, the dose of BCGwt and BCG.HIVA^222^ inoculated to newborn mice (2 × 10^6^ cfu) was 10-fold higher, as advised by the European Pharmacopoeia for the safety testing of live vaccines, in comparison with the most usual inoculation dose in adult mice [29]. Importantly, no differences were observed between the vaccinated mice groups and the naive mice group. On the other hand, the body weight profile was similar in all mice groups and similar to mice provider company standard body weight curve. Furthermore, between week 0 and week 14, the body weight monitored in all vaccinated mice groups was found between the mean body weight curve in naïve mice and the MVA.HIVA group (see Figure 5). Until week 14, the MVA.HIVA group can be considered as a naive group, because it has not been vaccinated yet. It is also important to mention that no mice died during the trial. Only local adverse events were detected in one mice showing slight redness and induration, which disappeared after several weeks. When analyzed by histopathology at necropsy, 17 weeks later, no severe lesion was observed.

## 4. Discussion

Despite the progress made on prevention of mother-to-child HIV-1 transmission, the development of a safe, effective, and affordable vaccine against HIV and TB at the earliest time after birth to prevent breast milk HIV transmission and childhood tuberculosis is still a great challenge. In the present study, we have evaluated the influence of age and immunization routes for induction of HIV-1 and *M. tuberculosis*-specific immune responses after BALB/c mice immunization with BCG.HIVA^222^ prime and MVA.HIVA boost. We have observed (i) enhanced specific T-cell induction responses in adult and newborn mice by using BCG.HIVA^222^ priming compared with BCG wild-type priming; (ii) higher frequencies and quality of the specific T-cell responses in adult mice immunized with BCG.HIVA^222^ intradermally compared with subcutaneously; (iii) that the BCG-specific immune responses were higher in newborn mice than adult mice; (iv) that among adult mice the BCG-specific immune responses were lower in mice primed with BCG.HIVA^222^ than BCG wild type; (v) that the BCG.HIVA^222^ prime and MVA.HIVA boost regimen is safe and immunogenic in newborn mice. Here, we have used the BCG.HIVA^222^ strain previously constructed by our group. This rBCG stably expresses the HIVA immunogen from the episomal pJH222HIVA plasmid. The HIVA gene was fused to the *M. tuberculosis* nucleotides coding for the 19-kDa lipoprotein signal sequence, and the HIVA gene expression was under the control of *Mycobacteria spp*. *α*-antigen promoter. 

In murine and nonhuman primates studies, we and others have shown that rBCG elicited antibody, and cell-mediated responses against HIV-1 and simian immunodeficiency virus antigens [30–34]. In fact, only a small proportion of these animal studies used rBCG strains in heterologous prime-boost regimens. Ami et al. [35] have demonstrated that macaques vaccinated with rBCG expressing SIV *gag* and boosted with replication defective poxvirus-SIV gag, elicited effective protective immunity against mucosal challenge with SHIV KS661c. There is evident data showing that rBCG is a good priming vector in heterologous prime-boost vaccination regimens with attenuated virus or recombinant proteins to enhance specific T-cell responses [14, 36–38]. In TB vaccine human trials, McShane et al. [37] have demonstrated that vaccination with MVA expressing Ag85 boosts preexisting antimycobacterial immune responses induced either by environmental mycobacteria or BCG vaccination. Hovav et al. [39] have explored novel priming immunogens that might be used in heterologous immunization regimens. They have shown that priming with recombinant *Mycobacterium smegmatis* expressing HIV-1 gp120 protein induced a cellular immune response that is biased toward memory CD8^+^ T cells and that can expand dramatically on reexposure to an HIV-1 envelope antigen. Our laboratory, in collaboration with Tomáš Hanke's laboratory have shown in BALB/c mice that the inclusion of BCG.HIVA^222^ in a heterologous prime-boost regimen can both prime novel and boost preexisting HIV-1 specific T-cell immune responses elicited by MVA.HIVA [14]. 

Many studies have compared the immune responses to foreign antigens delivered by rBCG inoculated by different routes; however, comparisons are difficult as doses, BCG strains, mycobacterial expression vectors, *in vivo* plasmid stability, promoters to regulate gene expression, levels of heterologous protein expression, and antigen localization are different. Several authors have emphasized the mucosal route of administration of rBCG. Lagranderie et al. [40] found that intrarectal immunization of mice with rBCG-SIV_mac251_ induced higher intestinal IgA responses than oral or nasal immunization. Kawahara et al. [41] examined a combined vaccination strategy in guinea pigs for enhancement of HIV-1-specific immune responses. They found that combined inoculation by rectal and intradermal routes effectively enhanced the levels of humoral and cellular immune responses against HIV-1. Promkhatkaew et al. [42] have explored the influence of immunization routes after adult BALB/c mice immunization with rBCG/HIV-1 gagE priming and rDIs/HIV-1 gagE boosting. They found higher CTL activity levels after two months subsequent to vaccination when mice were primed with rBCG subcutaneously and boosted with rDIs intravenously compared with priming and boosting intradermally. However, after seven months subsequent to vaccination, they found similar CTL activity levels when mice were primed with rBCG S.C. and boosted with rDIs i.v. or intradermally. In the current study, BCG.HIVA^222^ prime- and MVA.HIVA boost-elicited HIV-1-specific CD8^+^ T-cells exhibited effector functions such as production of IFN-*γ* and TNF-*α*, and such HIV-1-specific T-cell responses were higher in adult than in newborn mice. The inclusion of BCG.HIVA^222^ in a heterologous prime-boost regimen consistently enhanced and improved the frequency, quality, and durability of the generated HIV-1-specific responses in adult and newborn mice. This improvement was observed by the detection of the highest bifunctional HIVA-specific T-cell responses and higher specific cytolytic activity in the mice that received BCG.HIVA^222^ versus BCG wild type. Among adult mice, the intradermal inoculation of BCG.HIVA^222^ induced higher frequencies and quality of the specific HIV-1 immune responses versus the subcutaneous route. These results would be in accordance with the current recommended route of inoculation of BCG in infants. 

There are really few reports in the literature describing the safety and immunogenicity of rBCG expressing HIV antigens in neonatal mice and neonatal nonhuman primates. Ranganathan et al. [43] have evaluated the immunogenicity in neonatal mice of three different recombinant attenuated Mtb. strains expressing an HIV envelope. They showed that single-dose immunization in neonatal mice with ΔlysA ΔsecA2 Mtb strain expressing HIV Env rapidly generated HIV-1- and Mtb-specific T-cell immune responses. In the present study, we have shown in newborn mice that BCG.HIVA^222^ prime and MVA.HIVA boost increased the frequencies of specific CD8^+^ T cells producing IFN-*γ* or TNF-*α*. Such vaccine regimen also induced the highest proportion of HIV-1-specific bifunctional cells and the specific cytolytic activity. We have observed in newborn mice a lower level of HIV-1-specific T-cell immune responses compared with adult mice. Rosario et al. [44] have assessed the immunogenicity of the BCG.HIVA^222^ prime and MVA.HIVA boost regimen in newborn *Rhesus macaques*. They also observed that the HIV-1-specific responses induced in infants were lower compared with adult animals. On the other hand, we suggest that additional experiments should be performed in newborn mice inoculating the rBCG expressing HIV antigens by different routes, because the route of neonatal vaccination may confer different levels of immune activation, which may affect the efficacy of the vaccine. 

Here, the vaccination with BCG wild-type and BCG.HIVA^222^ strains induced strong BCG-specific responses in adult and newborn mice. Studies in neonatal mice have indicated that immune responses at birth are often biased towards the Th2 type and defective in the Th1 type, the central defense mechanism against intracellular pathogens. However, it has been described that BCG vaccination induces a potent Th1-type immune response at birth in humans and in mice [45–48].

The challenge for neonatal vaccinology is thus to develop, and promote at a global level, vaccines that could be safely administered soon after birth and would be effective after one or two early doses. According to our knowledge, no reports have been published about safety of rBCG-based HIV vaccine in neonatal mice. Rosario et al. [44] have demonstrated that BCGHIVA^401^ followed by two doses of MVA.HIVA in *Rhesus macaques* was safe, not associated with systemic reactions, and the local adverse events detected were considered to be consistent with a predicted response to the BCG vaccine administration, similar to that observed in human neonates. In the present study, we have demonstrated in neonatal mice (7 days old) that BCG.HIVA^222^ prime and MVA.HIVA boost regimen was safe. We observed only in one mouse a local reaction and induration, and no systemic changes were observed after necropsy. 

In conclusion, we tested the safety and immunogenicity of two candidate HIV-1 vaccines, BCG.HIVA^222^ and MVA.HIVA, in newborn mice using the prime-boost regimen. On the other hand we tested the influence of route inoculation among adult mice. We found the vaccines safe but less immunogenic for T cells in newborn mice than when administered to adult animals. In adult mice we found that the intradermal route gave higher frequencies and quality of the specific HIV-1 T-cell responses compared with the subcutaneous route. Given the urgent and global need for safe, effective, and affordable HIV and TB vaccines for infants and the demonstrated capability to produce and administer live mycobacterial vaccines on a large scale, BCG.HIVA^222^ prime and MVA.HIVA boost might be an attractive platform for a human neonatal vaccine for prevention of tuberculosis and mother-to-child breast milk transmission of HIV-1.
